# MTAS-MENA: adapting the Stroke Access Barrier Index (SABI) to enhance mechanical thrombectomy access in the Middle East and North Africa region

**DOI:** 10.3389/fneur.2026.1727476

**Published:** 2026-02-19

**Authors:** Ossama Yassin Mansour, Kaiz S. Asif, Violiza Inoa, Farid Aladham, Ibrahim Alnaami, Hosam Maher Al-Jehani, Abdulrahman Alshamy, Faisal Alghamdi, Ozlem Aykac, Mohamed Hamdy, Hany Hamadani, Mehdi Farhodi, Mahmoud Galal, Mohamed Ghorbani, Erdem Gurkas, Mohamed Alaa Habib, Nadia Hammami, Tamer Hassan, Farouk Hassan, Syed I. Hussain, Yahia Imam, Seby John, Wael Khalifa, Amina El Khamlichi, Amr Mahmoud, Ahmed Ossama, Mostafa Mahmoud, Ehab S. Mohamed, Nada Nasr, Atilla Ozcan Ozdemir, Umair Rashid, Salma Said, Abdulmonem Saied, Maher Saqqur, Khalid Sobh, Ryna Then, Gillian L. Gordon Perue, Mohammed Wasay, Hany Zaki Eldeen, Fawaz Al-Mufti, Santiago Ortega-Gutierrez, Sheila Martins, Dileep R. Yavagal, Ashfaq Shuaib

**Affiliations:** 1 Stroke and Neurointervention Center, Alexandria University, Alexandria, Egypt; 2 Department of Neurology, Ascension Medical Group Illinois, Chicago, IL, United States; 3 Department of Neurology, Semmes-Murphey Neurologic and Spine Clinic, Memphis, TN, United States; 4 Department of Neuroradiology, Amman Specialized IR Center, Amman, Jordan; 5 Department of Neurosurgery, College of Medicine, King Khalid University, Abha, Saudi Arabia; 6 Department of Neurosurgery, Imam Abdulrahman AL Faisal University, Dammam, Saudi Arabia; 7 Department of Neuroradiology, King Abdulaziz Medical City, Jeddah, Saudi Arabia; 8 Department of Interventional Neuroradiology, King Abdullah Medical City, Makkah, Saudi Arabia; 9 Department of Neurology, Eskisehir Osmangazi University, Eskisehir, Türkiye; 10 Department of Neuroradiology, Ain Shams University, Cairo, Egypt; 11 Department of Neurology, Ibn Al Nafees Hospital, Manama, Bahrain; 12 Department of Neurology, Tabriz University of Medical Sciences, Tabriz, Iran; 13 Department of Neurology, Al-Azhar University, Cairo, Egypt; 14 Department of Neurosurgery, Tehran University, Tehran, Iran; 15 Department of Neurology, Dr. Lutfi Kirdar City Hospital, Istanbul, Türkiye; 16 AinShams University, Unit, Cairo, Egypt; 17 Department of Interventional Neuroradiology, Institute National de Neurology, Tunis, Tunisia; 18 Department of Neuroradiology, Cairo University, Cairo, Egypt; 19 Department of Neurology, Neurological Institute Cleveland Clinic Abu Dhabi, Abu Dhabi, United Arab Emirates; 20 Department of Stroke and Vascular Neurology, Weill Cornell Medicine, Doha, Qatar; 21 Department of Neurology, Armed Forces Medical Complex in Maadi, Cairo, Egypt; 22 Interventional Neuroradiology, Centre Hospitalier Universaitaire IBN Sina de Rabat, Rabat, Morocco; 23 Department of Neurology, Tanta University, Tanta, Egypt; 24 Department of Neuroradiology, Lahore General Hospital, Lahore, Pakistan; 25 Department of Interventional Neuroradiology, Military Tripoli General Hospital, Tripoli, Libya; 26 Department of Neurology, University of Toronto, Ontario, Canada; 27 Department of Neurology, Cooper Medical School of Rowan University, Camden, NJ, United States; 28 Department of Neurology, University of Miami Miller School of Medicine, Miami, FL, United States; 29 Department of Clinical Neurology, Aga Khan University, Karachi, Pakistan; 30 Department of Neurology, New York Medical College, Valhalla, NY, United States; 31 Department of Neurology, University of Iowa, Iowa City, IA, United States; 32 University Federal of Rio Grande do Sul, Hospital de Clínicas de Porto Alegre, Porto Alegre, Brazil; 33 Department of Neurology, University of Miami Miller School of Medicine, Miami, FL, United States; 34 Department of Medicine, University of Alberta, Edmonton, AB, Canada

**Keywords:** access barriers, health disparities, healthcare systems, mechanical thrombectomy, MENA region, stroke

## Abstract

**Background and purpose:**

We developed and preliminarily validated the Stroke Access Barrier Index (SABI), adapted from the global MTAS framework, then applied it to assess potential barriers to mechanical thrombectomy access in the MENA region.

**Methods:**

Cross-sectional survey with embedded instrument development and validation study. We surveyed 352 stroke and neurointerventional facility directors across the Middle East and North Africa (MENA) region, defined as 22 countries/territories, with responses obtained from 17 countries (March–June 2024), receiving 102 responses (29%). The SABI tool evaluates 12 attributes scored 0-3, yielding a total score from 0-36. Emergency medical services (EMS) utilization and health literacy were key measures.

**Results:**

The median SABI score for the MENA region was 18.5 (IQR: 10.0–24.0; 95% CI: 16.8–20.2), significantly lower (*p* < 0.001) than the global median of 22.0 (IQR: 14.0-28.0; 95% CI: 21.3–22.7). High-income countries scored 24.0, while low-income countries scored 10.0. Physical barriers (median: 4.5/9.0) and sociocultural barriers (median: 4.0/9.0) were most prominent. EMS utilization (median: 1.0) and health literacy (median: 1.0) were consistently low across all income levels. Urban facilities (median: 20.5) substantially outperformed rural facilities (median: 13.0, *p* < 0.001).

**Conclusions:**

The SABI provides an exploratory framework for identifying potential MT access barriers in the MENA region. While preliminary findings suggest substantial challenges (median score 18.5), these results require validation through prospective studies linking scores to actual MT utilization and patient outcomes. This initial assessment may guide hypothesis generation for future intervention studies.

## Introduction

Mechanical thrombectomy (MT) has transformed large vessel occlusion (LVO) stroke management, demonstrating a number needed to treat of 2.6 to reduce disability (1). Despite proven efficacy, MT utilization varies dramatically across the MENA region, with < 1% of eligible patients in low-income countries receiving MT compared to 15-22% in high-income countries (2, 3).

The MENA region encompasses diverse economic, healthcare, and cultural landscapes, spanning high-income Gulf Cooperation Council countries with advanced healthcare infrastructure to low-income nations with fragile healthcare systems often compromised by conflict and political instability. Recent regional studies evaluating stroke centers across the MENA region revealed that a small minority had continuous 24/7 mechanical thrombectomy capability, with substantial disparities in service availability between urban and rural areas (4–6).

The Mechanical Thrombectomy Access Score (MTAS) has provided a valuable global framework for assessing MT access barriers (7). However, when applied to the MENA region, MTAS revealed limitations in capturing region-specific challenges, particularly sociocultural factors such as gender-specific healthcare access issues, religious practices influencing healthcare-seeking behavior, and the impact of regional conflicts. To address these limitations, this study aimed to adapt the global MTAS framework for the MENA region by developing and applying the Stroke Access Barrier Index (SABI), a tool designed to systematically quantify region-specific barriers to mechanical thrombectomy access across physical, diagnostic/information, financial, and sociocultural domains. While MTAS provides valuable global insights, no existing tool adequately captures MENA-specific sociocultural barriers, including gender-based healthcare access disparities and ongoing conflict impacts.

The primary aims of this study were to (1) develop and validate the SABI tool adapted for the MENA context, and (2) apply this tool to assess current barriers to mechanical thrombectomy access across 17 MENA countries, identifying priority interventions based on domain-specific findings.

## Methods

### Survey design and development

For this study, we define “barriers” as modifiable or non-modifiable factors that impede timely access to mechanical thrombectomy services. The MTAS-MENA adaptation, utilizing the SABI methodology, was developed through a rigorous multi-step process building upon the global MTAS framework. First, a comprehensive literature review identified key barriers to MT access globally and within the MENA region specifically. Second, a panel of 15 stroke specialists and healthcare system experts from diverse MENA countries participated in a modified Delphi process to refine the domains and attributes. The 15 Delphi participants included stroke neurologists (*n* = 6), interventional neuroradiologists (*n* = 4), emergency medicine physicians (*n* = 3), and health policy experts (*n* = 2) from Egypt, Saudi Arabia, Turkey, UAE, and Jordan. Participants had a mean age of 48 years (SD = 8.2), 73% were male, and all had >10 years of experience in stroke care. This process resulted in a 12-attribute tool organized across four domains, each with three attributes scored from 0 (severe barrier) to 3 (no barrier). The survey instrument underwent pilot testing with 20 stroke specialists from five MENA countries (Egypt, Saudi Arabia, Turkey, Morocco, and Iran) to assess face and content validity. This pilot phase led to three key refinements: expanded sociocultural domain for gender-based barriers, adjusted EMS utilization thresholds (severe barrier: < 10% vs. moderate: 10–30%), and broadened telemedicine definitions to include informal physician consultations. Full pilot testing procedures are detailed in Supplementary Appendix S1.

After these revisions, the tool demonstrated good internal consistency (Cronbach's alpha = 0.82). Psychometric Validation: Psychometric validation included: (1) Content validity through expert panel review and pilot testing with Content Validity Index = 0.92 for individual items, (2) Internal consistency assessment using Cronbach's alpha (criterion: α > 0.70), (3) Inter-rater reliability using intraclass correlation coefficients for continuous scores and Cohen's kappa for categorical items, (4) Construct validity through exploratory factor analysis using principal component extraction with varimax rotation (Kaiser-Meyer-Olkin measure = 0.84, Bartlett's test *p* < 0.001). The four-factor solution explained 72% of total variance. Test-retest reliability was not assessed due to the cross-sectional design.

### Survey distribution and respondent selection

A cross-sectional survey was conducted targeting directors of stroke and NIR facilities across the Middle East and North Africa (MENA) region. For the purposes of this study, we defined MENA as comprising 22 countries and territories: Algeria, Bahrain, Egypt, Iran, Iraq, Israel, Jordan, Kuwait, Lebanon, Libya, Morocco, Oman, Pakistan, Palestine, Qatar, Saudi Arabia, Sudan, Syria, Tunisia, Turkey, United Arab Emirates (UAE), and Yemen. This definition follows the World Bank regional classification with the addition of Turkey, Iran, and Pakistan given their geographic proximity and shared healthcare system characteristics with the core MENA region.

Survey responses were obtained from 17 countries: Egypt, Algeria, Tunisia, Morocco, Sudan, Turkey, UAE, Saudi Arabia (KSA), Jordan, Bahrain, Kuwait, Iraq, Iran, Pakistan, Yemen, Qatar, and Syria. Five countries/territories did not provide survey responses: Israel, Lebanon, Libya, Oman, and Palestine. Non-response from these countries was attributable to inability to identify eligible facility directors through available professional networks (Israel, Palestine), ongoing political instability limiting survey distribution (Lebanon, Libya), or lack of response despite repeated invitations (Oman). Participants were identified through multiple sources, including national stroke society membership directories, hospital registries from ministries of health, regional neurology and interventional radiology professional networks, and academic medical center directories. Participants received no compensation for survey completion. The survey was distributed electronically via REDCap between March 15, 2024, and June 20, 2024.

The survey was distributed electronically via REDCap between March 15, 2024, and June 20, 2024. Of 352 directors invited, 102 provided complete responses (29% response rate). Response rates varied by country, ranging from 45% in Turkey to 15% in Yemen, with politically unstable regions showing lower participation. Non-responders received up to three reminder emails at two-week intervals. Non-responder analysis compared characteristics of responding vs. non-responding facilities using publicly available data. No significant differences were found in facility type (*p* = 0.34), urban/rural distribution (*p* = 0.28), or country income level (*p* = 0.42). Missing data (< 6% for any attribute) were handled using multiple imputation by chained equations (MICE) with 10 iterations, incorporating facility type, location, and country income as predictors. Sensitivity analyses comparing complete-case analysis (*n* = 96) with imputed results showed no meaningful differences in SABI scores (mean difference: 0.3 points, *p* = 0.67) or domain rankings.

### Stroke Access Barrier Index (SABI) components

SABI assesses 12 attributes across four domains, with each domain contributing equally (maximum 9 points) to the total score (maximum 36 points):

**Physical barriers (9 points):**

- EMS utilization: Proportion of stroke patients arriving via emergency medical services.
- Transportation infrastructure: Availability and quality of transportation options to MT centers.
- Access to MT centers: Geographic distribution and density of MT-capable facilities.

**Diagnostic/information barriers (9 points):**

- Stroke imaging availability: Access to CT/MRI and vascular imaging.
- ED stroke triage systems: Protocols for rapid identification and assessment.
- Telemedicine access: Availability of telestroke networks for remote assessment.

**Financial barriers (9 points):**

- Insurance coverage: Proportion of population with coverage for MT procedures.
- Device availability: Access to necessary thrombectomy devices and supplies.
- MT operator availability: Availability of trained neurointerventionalists.

**Sociocultural barriers (9 points):**

- Health literacy: Public knowledge about stroke symptoms and treatment.
- Cultural beliefs about stroke: Misconceptions that may delay care-seeking.
- Trust in healthcare providers: Willingness to accept recommended interventions.

Each attribute was scored using standardized criteria (Supplementary Table S1), with higher scores indicating fewer barriers. Domain-specific intraclass correlation coefficients ranged from 0.71 (sociocultural) to 0.82 (financial), with detailed values reported in Supplementary Table S1.

### Data analysis

“Responses were aggregated by country and analyzed using descriptive statistics, with median SABI scores calculated with interquartile ranges and 95% confidence intervals. Regional scores were compared to global benchmarks using Mann-Whitney U test. Global benchmark data (median SABI: 22.0) were derived from the published MT-GLASS study (8), which surveyed 237 facilities across 56 countries using comparable methodology. Direct statistical comparisons should be interpreted cautiously given different sampling frames and response rates. Subgroup analyses were performed by income level, facility location, and specialty. Inter-rater reliability (Cohen's kappa = 0.76) and hierarchical clustering analysis were performed to identify country groups with similar barrier profiles. Statistical significance was set at *p* < 0.05 using SPSS version 28.0. Income level was entered as an ordinal variable (coded 1–4) in regression models with tests for linear trend. Sensitivity analyses using dummy variables (reference: high-income) yielded similar results. Missing data patterns were assessed using Little's MCAR test (*p* = 0.23, suggesting missing completely at random). Model assumptions were verified through residual plots (linearity), variance inflation factors (multicollinearity, all VIF < 3), and Durbin-Watson statistics (independence, DW = 1.92). Multivariable linear regression models were constructed with SABI scores as the dependent variable and country income level (four categories) and facility location (urban/rural) as independent variables. Adjusted analyses controlled for respondent specialty and facility type. Models were tested for multicollinearity (VIF < 5) and assumptions of linearity. Full methodological details, including intervention feasibility ratings and extended pilot testing modifications, are available in Supplementary Appendix S1. Full methodological details, including intervention feasibility ratings and extended pilot testing modifications, are available in Supplementary Appendix S1. Sample size calculation indicated that 96 responses would provide 90% power to detect a difference of 3.5 points in total SABI score between MENA and global benchmarks, assuming α = 0.05 and standard deviation of 8.0.

### Sensitivity analyses

We conducted three sensitivity analyses to assess the robustness of our findings: (1) variation of impact estimates with ±50% changes in key assumptions,(2) bootstrap confidence intervals for all domain scores using 1,000 iterations, and (3) alternative SABI domain weighting schemes including expert-derived weights and data-driven weights based on principal component analysis. Full sensitivity analyses are presented in Supplementary Table S8.

#### Sensitivity analysis methodology

Impact Estimate Sensitivity: We varied five key parameters by ±50% from base case values: MENA population, stroke incidence, LVO rate, current MT utilization, and target utilization. Monte Carlo simulation (10,000 iterations) generated confidence intervals assuming normal distributions for each parameter.

Bootstrap Confidence Intervals: We performed 1,000 bootstrap iterations sampling with replacement from the original dataset (*n* = 102) to generate 95% confidence intervals for all domain and attribute scores, stratified by income level.

Alternative Weighting Schemes: Five alternative weighting schemes were tested: (1) Expert-derived weights from Delphi panel rankings, (2) Data-driven weights using principal component analysis based on variance contribution, (3) Clinical priority weights emphasizing time-sensitive factors, (4) Infrastructure-focused weights prioritizing system capacity, and (5) Patient-centered weights emphasizing access barriers. Correlation coefficients and coefficients of variation assessed stability across schemes.

#### Ethical considerations

This study did not require formal ethical approval as it consisted of a survey sent to physicians to collect information about stroke services and mechanical thrombectomy infrastructure without gathering any patient data or protected health information. Nonetheless, electronic informed consent was obtained from all participating physicians prior to their completion of the survey, and all responses were anonymized during analysis to protect respondent confidentiality.

Study Limitations and Scope: This study presents an exploratory assessment tool for identifying MT access barriers. SABI has not yet been validated against actual MT utilization rates or patient outcomes. The tool's current validation includes internal consistency (α = 0.82) and inter-rater reliability (κ = 0.76), representing preliminary psychometric properties. A prospective validation study is currently being designed to correlate SABI scores with actual MT utilization rates and 90-day modified Rankin Scale outcomes. The validation will require 150 centers (power calculation: 80% power to detect correlation r ≥ 0.35, α = 0.05) with data collection planned over 18 months starting January 2025. Primary endpoints include correlation between baseline SABI scores and (1) MT utilization rates per 100,000 population and (2) proportion achieving mRS 0–2 at 90 days.

## Results

### Respondent characteristics

Of the 102 respondents, 61 (60%) were from urban centers, 27 (26%) from semi-urban areas, and 14 (14%) from rural facilities. Specialties included interventional neurology (36 respondents, 35%), stroke neurology (25, 25%), general neurology (20, 20%), interventional cardiology (11, 10%), and endovascular neurosurgery (10, 10%). The distribution of respondents by country is presented in Supplemental Table S2.

### SABI development and validation

The SABI tool development process involved a systematic literature review of 127 articles, followed by a three-round Delphi process with 15 experts from five MENA countries. Pilot testing with 20 specialists led to three key refinements: expansion of the sociocultural domain to include trust in healthcare providers (based on 85% consensus), adjustment of EMS utilization thresholds to reflect regional realities, and broadening of telemedicine definitions to include informal consultations. The final tool demonstrated good internal consistency (Cronbach's α = 0.82) with domain-specific alphas ranging from 0.71 (sociocultural) to 0.82 (financial). Inter-rater reliability was substantial (Cohen's κ = 0.76) with domain-specific ICCs from 0.71 to 0.82.

### SABI scores for the MENA region

**Figure 1:**
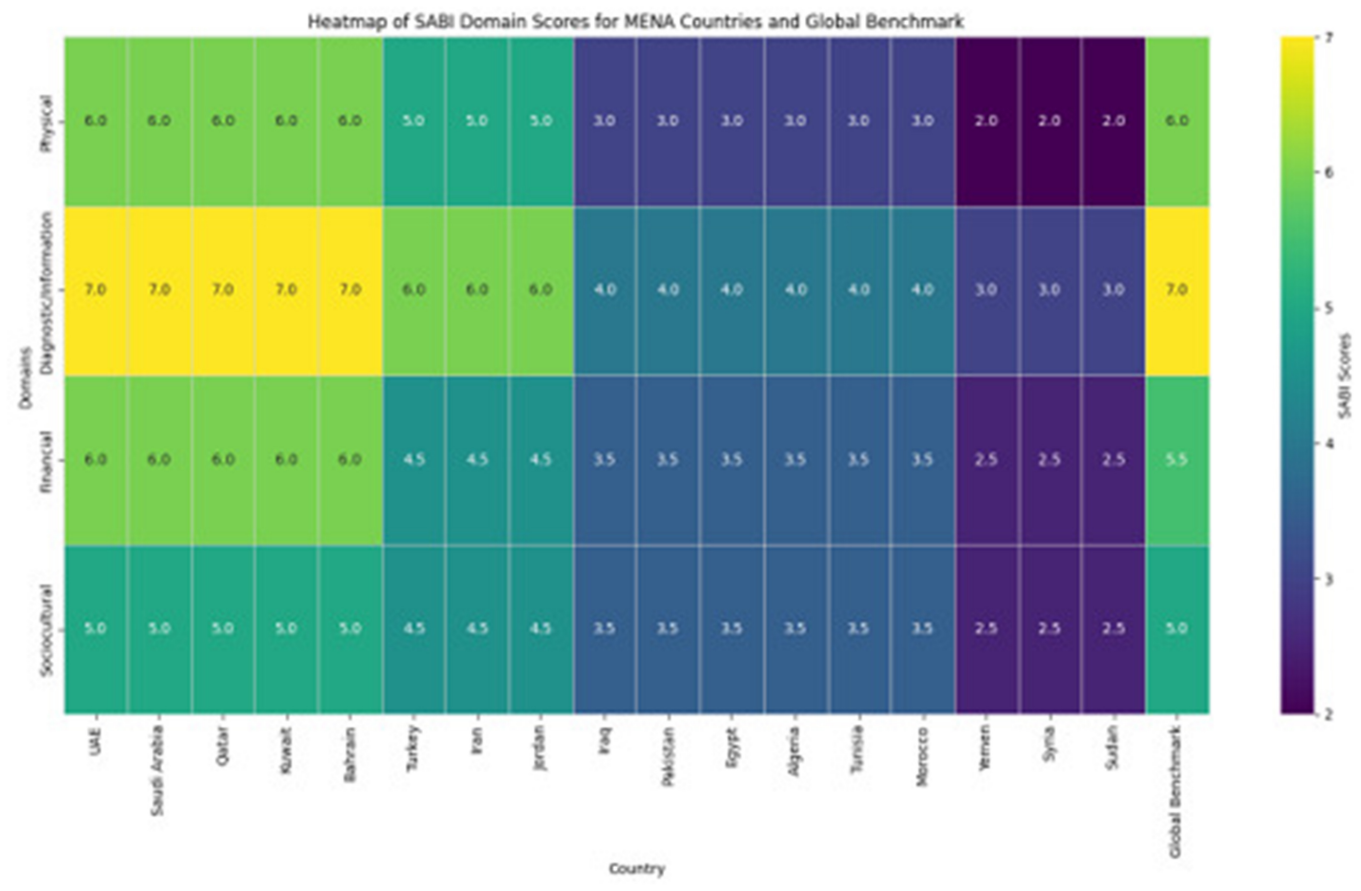
Heatmap of SABI domain scores across MENA countries compared to global benchmark. Color intensity represents score magnitude (darker = higher scores/fewer barriers). Note the consistently lower scores in physical and sociocultural domains across all MENA countries compared to global benchmarks. Countries ordered by total SABI score from highest (UAE, Saudi Arabia, Qatar: 24.0) to lowest (Sudan, Yemen: 10.0).

**Figure 2:**
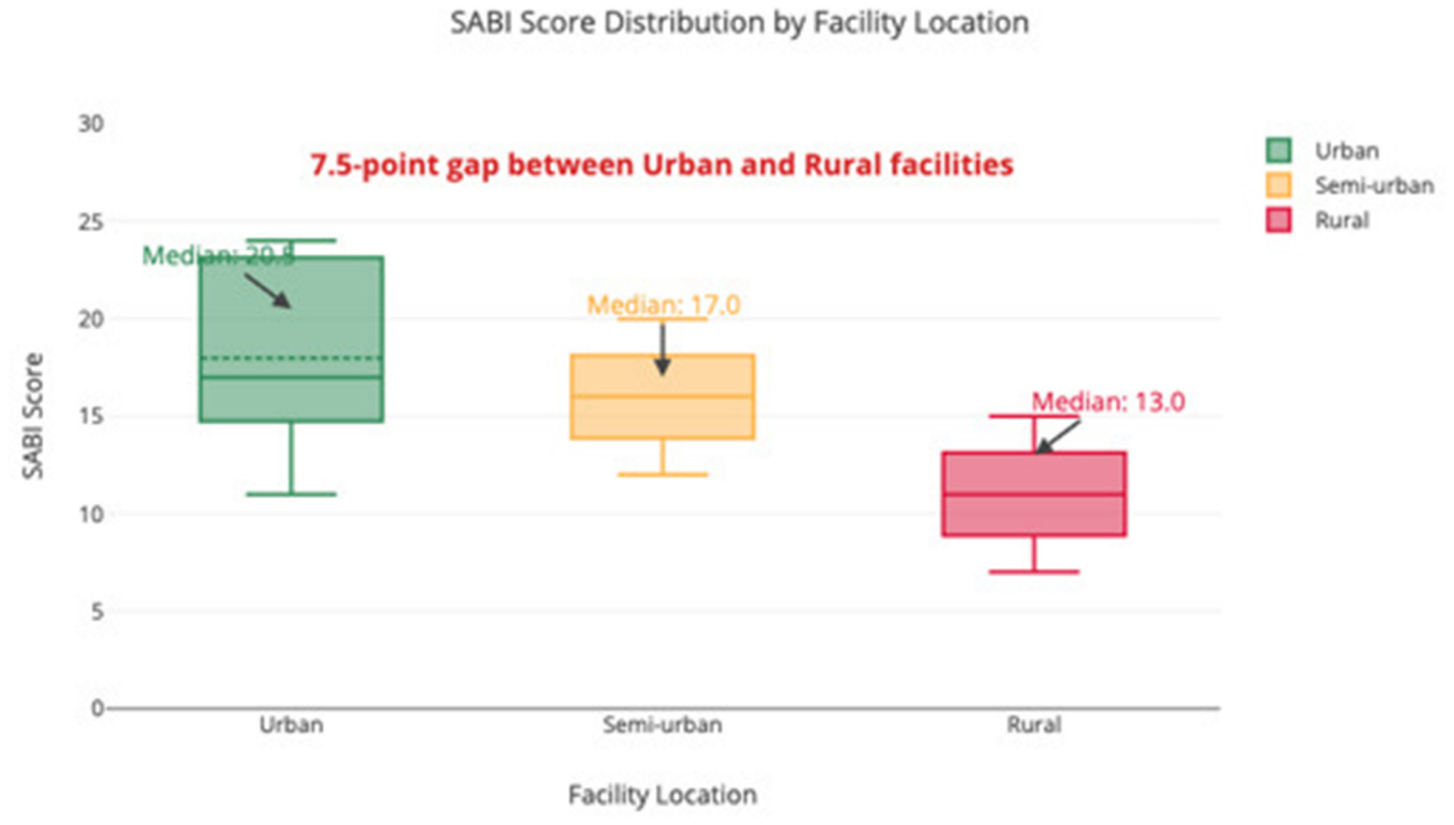
Box-and-whisker plot comparing SABI scores between urban and rural facilities across income levels. Boxes represent interquartile ranges with median lines; whiskers extend to 1.5 × IQR; dots indicate outliers. Urban facilities (n = 61, median: 20.5, IQR: 17.0–23.5) significantly outperformed rural facilities (n = 14, median: 13.0, IQR: 10.0–16.0), p < 0.001. The 7.5-point gap persists across all income strata, with transportation infrastructure showing the largest urban-rural difference (Δ = 1.5 points).

The median SABI score for MENA was 18.5 (IQR: 10.0–24.0; 95% CI: 16.8–20.2), significantly lower than the global median of 22.0 (IQR: 14.0-28.0; 95% CI: 21.3–22.7), *p* < 0.001 (Figure 1). Scores varied by income level: high-income 24.0, upper-middle-income 19.5, lower-middle-income 14.0, and low-income 10.0. Urban facilities (median: 20.5) scored significantly higher than rural facilities (median: 13.0, *p* < 0.001) (Figure 2).

### Domain-specific findings

Domain-specific analysis revealed significant variations across all four barrier categories (Table 1). Physical Barriers scored 4.5/9.0 (IQR: 4.0–5.0; 95% CI: 4.2–4.8), significantly lower than the global median of 6.0/9.0 (IQR: 5.5–6.5; 95% CI: 5.7–6.3), *p* < 0.001. EMS utilization was particularly low (median: 1.0; IQR: 0.5–1.5; 95% CI: 0.8–1.2), with only 30% reporting EMS use for >30% of stroke patients. Rural transportation infrastructure scored 0.5 vs. 2.0 in urban areas (*p* < 0.001).

**Table 1:** SABI domain scores by income level.

**Income level**	***n***	**Physical barriers**	**Diagnostic/information**	**Financial barriers**	**Sociocultural barriers**	**Total SABI score**
High-income	30	6.0 (5.5–6.5)	7.0 (6.5–7.5)	6.0 (5.5–6.5)	5.0 (4.5–5.5)	24.0 (22.3–25.7)
Upper-middle	28	5.0 (4.5–5.5)	6.0 (5.5–6.5)	4.5 (4.0–5.0)	4.5 (4.0–5.0)	19.5 (17.8–21.2)
Lower-middle	35	3.0 (2.5–3.5)^*^	4.0 (3.5–4.5)^*^	3.5 (3.0–4.0)^*^	3.5 (3.0–4.0)^*^	14.0 (12.3–15.7)^*^
Low-income	9	2.0 (1.5–2.5)^*^	3.0 (2.5–3.5)^*^	2.5 (2.0–3.0)^*^	2.5 (2.0–3.0)^*^	10.0 (8.2–11.8)^*^
**MENA overall**	102	4.5 (4.2–4.8)	6.0 (5.7–6.3)	4.0 (3.7–4.3)	4.0 (3.7–4.3)	18.5 (16.8–20.2)
**Global benchmark**	-	6.0 (5.7–6.3)^†^	7.0 (6.7–7.3)^†^	5.5 (5.2–5.8)^†^	5.0 (4.7–5.3)^†^	22.0 (21.3–22.7)^†^

Values are median (95% CI); ^*^p < 0.001 vs. high-income; ^†^*p* < 0.001 vs MENA overall.

Note: SABI = Stroke Access Barrier Index; CI, Confidence Interval; MENA, Middle East and North Africa.

Each domain has a maximum score of 9.0, with higher scores indicating fewer barriers.

^*^p-values represent comparison with global median using Mann-Whitney U test.

Diagnostic/Information Barriers scored 6.0/9.0 (IQR: 5.5–6.5; 95% CI: 5.7–6.3), significantly lower than the global median of 7.0/9.0 (IQR: 6.5–7.5; 95% CI: 6.7–7.3), *p* = 0.02. Stroke imaging was available in high/upper-middle-income countries (median: 2.5; IQR: 2.0–3.0) but limited in lower-income countries (median: 1.0; IQR: 0.5–1.5), *p* < 0.001 (Supplementary Table S3). Only 22% of facilities reported established telestroke networks.

Financial Barriers scored 4.0/9.0 (IQR: 3.5–4.5; 95% CI: 3.7–4.3), significantly lower than the global median of 5.5/9.0 (IQR: 5.0–6.0; 95% CI: 5.2–5.8), *p* < 0.001. Insurance coverage was limited (median: 1.0; IQR: 0.5–1.5), with < 30% of MT procedures fully reimbursed in 12/17 countries (Supplementary Table S3). Device availability varied from 2.5 in high-income to 0.5 in low-income countries (*p* < 0.001).

Sociocultural Barriers scored 4.0/9.0 (IQR: 3.5–4.5; 95% CI: 3.7–4.3), significantly lower than the global median of 5.0/9.0 (IQR: 4.5–5.5; 95% CI: 4.7–5.3), *p* = 0.003. Health literacy was low (median: 1.0; IQR: 0.5–1.5), with 75% reporting < 50% of population could identify stroke symptoms. Cultural beliefs and trust in healthcare providers (both median: 1.5; IQR: 1.0–2.0) showed regional variations (Supplementary Table S3).

Gender-Specific and Conflict-Related Barriers: Gender-specific barriers emerged as a critical theme within the sociocultural domain. Female respondents reported additional challenges including restricted mobility (68% of facilities), preference for female providers (45%), and family decision-making requirements (52%). Women showed 20-30% lower EMS utilization rates compared to men across surveyed countries. Conflict-affected countries (Syria, Yemen, Iraq) demonstrated consistently lower scores across all domains, with particularly severe impacts on device availability (median: 0.5 vs. 1.5 in stable countries, *p* < 0.001) and healthcare workforce availability (median: 0.5 vs. 1.5, *p* < 0.001).

Sensitivity analyses demonstrated that SABI scores remained stable across alternative weighting schemes (coefficient of variation < 15%). Impact estimates showed wider uncertainty ranges (5,000–25,000 disabilities prevented) when key assumptions were varied by ±50%. Bootstrap confidence intervals confirmed the statistical significance of all primary findings (Supplementary Table S8).

### Country-specific analysis and cluster patterns

**Figure 3:**
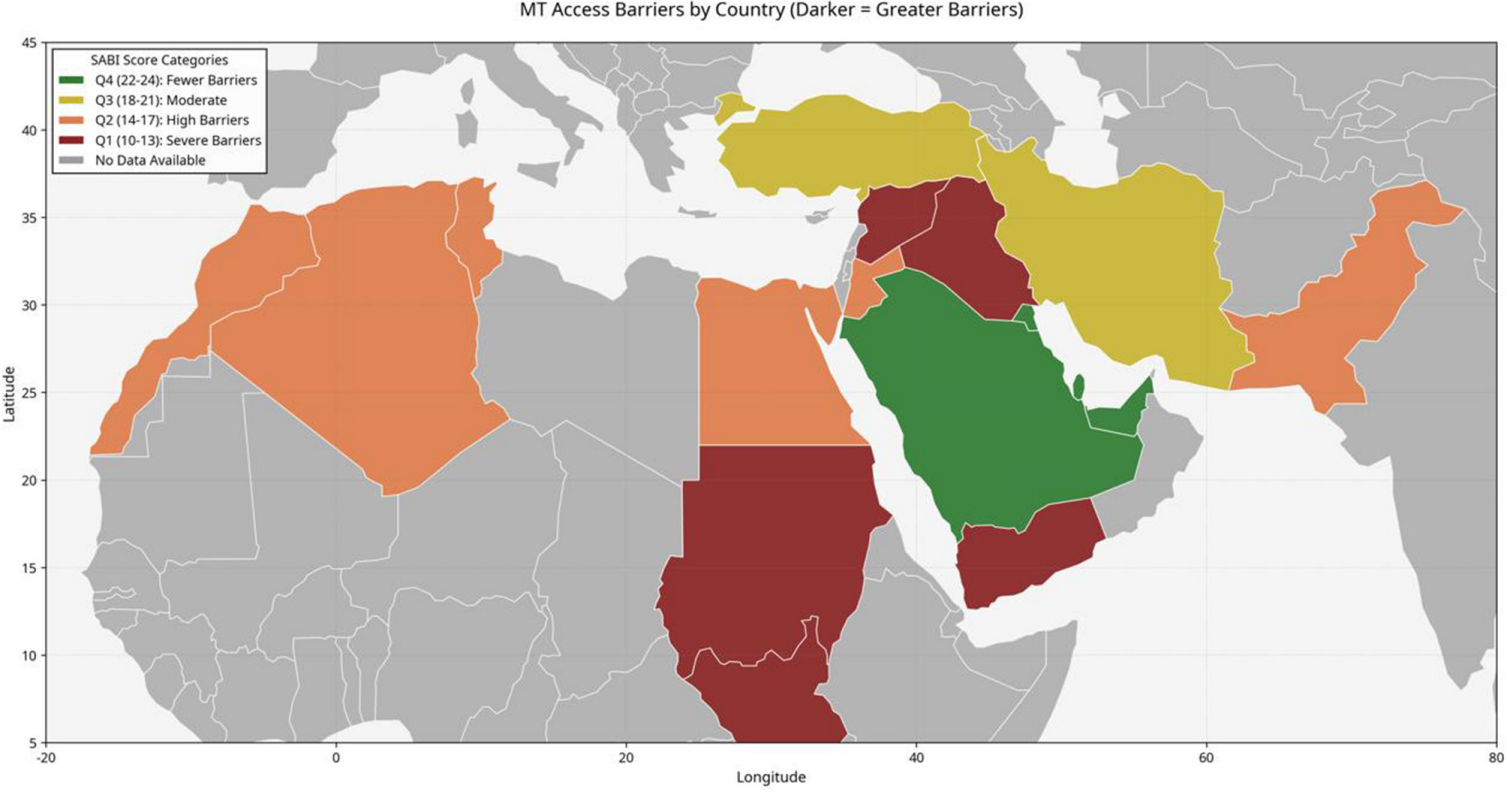
Choropleth map showing geographic distribution of total SABI scores across the MENA region. Countries colored by SABI score quartiles: Q1 (10–13): red, Q2 (14–17): orange, Q3 (18–21): yellow, Q4 (22–24): green. Grey indicates no data available. The map demonstrates clear clustering of high scores in Gulf Cooperation Council countries and progressively lower scores in conflict-affected regions. Inset shows correlation between SABI scores and country income level (R = 0.82, *p* < 0.001).

Hierarchical clustering analysis identified three distinct country clusters with similar barrier profiles (Figure 3), with substantial variation in individual country scores (Table 2).

**Table 2:** Country-level SABI scores and key barrier indicators.

**Country**	**Income level**	**Total SABI score (95% CI)**	**Physical domain**	**Diagnostic domain**	**Financial domain**	**Sociocultural domain**	EMS utilization^*^	Insurance coverage^*^	Health literacy^*^
**High-income countries**									
UAE	High	24.0 (22.5–25.5)	6.0	8.0	7.5	5.0	1.5	2.5	1.5
Saudi Arabia	High	24.0 (22.5–25.5)	6.0	8.0	7.5	5.0	1.5	2.5	1.5
Qatar	High	24.0 (22.5–25.5)	6.0	8.0	7.5	5.0	1.5	2.5	1.5
**Upper-middle income countries**									
Turkey	Upper–middle	20.0 (18.5–21.5)	5.0	7.5	4.5	4.5	1.5	1.5	1.0
Iran	Upper–middle	19.5 (18.0–21.0)	5.0	6.5	4.5	4.5	1.5	1.0	1.5
**Lower-middle income countries**									
Egypt	Lower–middle	14.0 (12.5–15.5)	3.5	4.5	3.5	3.0	1.0	0.5	1.0
Pakistan	Lower–middle	14.0 (12.5–15.5)	3.0	4.5	3.5	3.5	0.5	0.5	1.0
**Low-income countries**									
Sudan	Low	10.0 (8.5–11.5)	2.0	3.0	2.5	2.5	0.5	0.5	0.5
Yemen	Low	10.0 (8.5–11.5)	2.0	3.0	2.5	2.5	0.5	0.5	0.5
**Regional summary**									
MENA median	–	18.5 (16.8–20.2)	4.5	6.0	4.0	4.0	1.0	1.0	1.0
Global median	–	22.0 (21.3–22.7)^†^	6.0^†^	7.0^†^	5.5^†^	5.0^†^	2.0^†^	2.0^†^	2.0^†^

^*^Selected critical attributes shown; full attribute scores in Supplementary Table S3 ^†^*p* < 0.001 for comparison with MENA median.

Domain scores range 0–9; attribute scores range 0–3; higher scores indicate fewer barriers.

Note on Data Completeness: Response rates for individual attributes ranged from 94% to 100%. Missing data (< 6% for any attribute) were handled using multiple imputation with 10 iterations. Sensitivity analyses comparing complete case analysis with imputed results showed no significant differences in main findings (all *p* > 0.20).

Hierarchical clustering identified three distinct country clusters: Cluster 1 (High-Performing Gulf Countries): UAE, Saudi Arabia, Qatar, Kuwait, and Bahrain - median SABI score 24.0, strong financial support (7.5/9.0) and diagnostic infrastructure (8.0/9.0), but low EMS utilization (1.5) and health literacy (1.5). Cluster 2 (Middle-Income Transitioning Systems): Turkey, Iran, Jordan, Algeria, Tunisia, and Morocco - median SABI score 17.5, with diagnostic capabilities (6.0/9.0) partially offsetting moderate physical (4.5/9.0) and financial barriers (4.0/9.0). Cluster 3 (Resource-Limited and Conflict-Affected): Egypt, Pakistan, Iraq, Syria, Sudan, and Yemen - median SABI score 12.0, with severe barriers across all domains, particularly physical (2.5/9.0) and financial (3.0/9.0). Table with detailed attribute-level scores available in Supplementary Table S3.

### Robustness of findings

Sensitivity analyses confirmed the stability of our primary findings. SABI scores showed high correlation (*r* = 0.94–0.98) across all alternative weighting schemes, with coefficient of variation < 15%. Impact estimates demonstrated wider uncertainty when assumptions were varied, ranging from 5,000 disabilities prevented (conservative scenario with all parameters at −50%) to 25,000 (optimistic scenario with all parameters at +50%). Bootstrap analysis confirmed that differences between income groups remained statistically significant across 1,000 iterations (all *p* < 0.001).

## Discussion

This study represents the first comprehensive development and application of the Stroke Access Barrier Index (SABI) across 17 countries in the MENA region, revealing significant but preliminary insights into barriers to mechanical thrombectomy access. With a median SABI score of 18.5 (95% CI: 16.8–20.2), substantially below the global median of 22.0, our findings suggest critical gaps that warrant further investigation and targeted intervention.

The development of SABI addressed limitations in the global MTAS framework, particularly in capturing MENA-specific sociocultural challenges. SABI's 12 attributes across four domains enable nuanced understanding of barriers, facilitating targeted interventions. The tool demonstrated high internal consistency (Cronbach's α = 0.82) and inter-rater reliability (Cohen's κ = 0.76), establishing it as a robust instrument for measuring MT access barriers. Further validation with clinical outcomes data will be essential to establish predictive validity. SABI's validation demonstrates strong psychometric properties essential for reliable barrier assessment. The high internal consistency (Cronbach's α = 0.82) and inter-rater reliability (Cohen's κ = 0.76) establish measurement stability across diverse healthcare settings. However, establishing predictive validity through correlation with actual MT utilization rates and patient outcomes remains crucial for confirming SABI's clinical relevance. Future validation studies linking SABI scores to modified Rankin Scale outcomes will strengthen its utility as a healthcare planning instrument.

The disparity in SABI scores between high-income (24.0) and low-income countries (10.0) reflects the profound impact of economic resources on stroke infrastructure. High-income Gulf countries have established advanced stroke centers yet face challenges in pre-hospital systems and public awareness. Their high financial scores (7.5/9.0) contrast with low-income countries (2.5/9.0) where limited healthcare financing poses significant barriers. Middle-income countries like Turkey and Iran demonstrate that strategic resource allocation can partially overcome financial limitations through strong diagnostic infrastructure (6.5–7.5/9.0). However, comprehensive improvement requires multi-faceted approaches beyond technological investments. The rural-urban divide (urban: 20.5 vs. rural: 13.0) is concerning given that 42% of MENA population lives in rural areas, with transportation infrastructure showing the most pronounced gaps (2).

**Physical barriers:** The consistently low EMS utilization (median: 1.0) represents a critical bottleneck. Previous studies show EMS use for stroke in MENA ranges from 1.8% in Morocco to 65.1% in Qatar (9–14).

- Priority interventions should focus on public awareness campaigns using culturally appropriate messaging, EMS stroke training programs for rapid recognition and triage, and geographic optimization of MT centers using hub-and-spoke models (15, 16).

**Diagnostic/information barriers:** Limited telestroke adoption (22% of facilities) represents a missed opportunity. Globally, only 7.5% of telestroke networks exist in LMICs vs. 74.1% in HICs (17). Key interventions include ED physician certification programs in stroke management, standardized imaging protocols for resource-limited settings, and regional telestroke networks linking high- and low-resource centers (18).

- Financial Barriers: Limited insurance coverage (median: 1.0) affects both patient access and institutional capacity, with < 30% of procedures fully reimbursed in most countries. Essential interventions include regional purchasing consortia for device cost reduction, national insurance reform to include MT in essential benefits, and cross-subsidization programs in dual-system healthcare environments (19).

**Sociocultural barriers**: Poor health literacy (median: 1.0) was widespread, with 75% reporting < 50% of population could identify stroke symptoms. Cultural beliefs and gender-specific barriers further contribute to delays. Effective interventions include faith-based education programs leveraging religious networks, school-based stroke awareness programs, and gender-sensitive approaches addressing women's unique barriers (20, 21).

Gender-specific barriers were prominent across the region, with women facing unique challenges including restricted mobility, preference for female providers, and financial dependence affecting healthcare decisions. These findings align with previous studies showing 20–30% lower EMS utilization among women in several MENA countries (3, 9, 22).

These findings provide evidence-based targets for national stroke programs and international development agencies prioritizing healthcare investments. The SABI framework enables systematic monitoring of intervention impact over time.

Several limitations warrant consideration in interpreting these findings. First, the 29% response rate, while comparable to similar multinational surveys and representing the largest systematic assessment of MT barriers in MENA (102 facilities, 17 countries), may introduce selection bias, particularly in conflict-affected regions where response rates were lowest (15–20%). Notably, the lower participation from conflict-affected countries (Syria: 16%, Yemen: 15%, Iraq: 20%) likely results in a relatively optimistic assessment of MT availability across the region, as facilities in these settings that did not respond may face even more severe barriers than those captured in our data. Consequently, the true regional SABI median may be lower than the 18.5 reported, and the disparities between stable and conflict-affected settings may be underestimated. Second, the cross-sectional design captures barriers at a single timepoint without assessing temporal changes or seasonal variations. Third, equal domain weighting in the SABI tool may oversimplify complex interactions between barriers, though sensitivity analyses with alternative weighting schemes showed robust results (*r* = 0.94–0.98). Fourth, and critically, validation against actual MT utilization rates and clinical outcomes remains pending, limiting the tool's current clinical applicability. Fifth, surveying only facility directors may miss important perspectives from emergency medical services personnel, frontline healthcare workers, and patients themselves. Sixth, some sociocultural assessments rely on clinician perceptions rather than direct patient data, potentially underestimating the true impact of these barriers. Finally, the tool requires adaptation and validation before use in other regions. The wide confidence intervals for impact estimates (5,000–25,000 disabilities prevented) reflect substantial uncertainty in our projections and underscore the need for prospective validation studies.

To advance the Stroke Access Barrier Index (SABI), MENA-SINO has launched a dynamic geomapping tool (https://mena-sino.com/sabi-tool/) that integrates validation studies and future research avenues to enhance mechanical thrombectomy (MT) access across the MENA region. The platform conducts a prospective cohort study across 50 centers to correlate SABI scores with MT utilization, assesses intervention impacts (e.g., EMS training), links SABI to patient outcomes (modified Rankin Scale), and defines score thresholds via expert consensus. Real-time geomaps visualize SABI attributes and outcomes, guiding interventions in settings like Egypt (SABI: 14.0) and Qatar (SABI: 24.0). Future efforts include longitudinal SABI assessments to evaluate policy reforms, patient-centered barrier studies, targeted intervention implementations, SABI adaptation for other regions, and geospatial integration to optimize MT center placement and transportation systems.

Exploratory modeling suggests that addressing identified barriers could potentially increase MT utilization, though these preliminary estimates carry substantial uncertainty (see Supplementary Appendix S2 for calculations and sensitivity analyses). Pilot interventions focusing on the most severe barriers identified in this study should be prioritized, particularly those addressing EMS utilization and health literacy, which consistently scored low across all countries.

## Conclusions

The Stroke Access Barrier Index provides a comprehensive framework for assessing MT access barriers in the MENA region. With a median score of 18.5, significantly below global benchmarks, the region faces substantial challenges in providing equitable access to this life-saving intervention. Physical and sociocultural barriers are particularly pronounced, with EMS utilization and health literacy representing critical targets for improvement. Addressing these barriers could transform stroke care for 450 million MENA residents, particularly the 42% in underserved rural areas.

## Data Availability

The datasets presented in this study can be found in online repositories. The names of the repository/repositories and accession number(s) can be found below: The anonymized dataset supporting this study's findings is available from the corresponding author (Dr. Ossama Yassin Mansour, yassinossama@yahoo.com) upon reasonable request, subject to approval from participating institutions and compliance with data protection regulations. The SABI survey instrument and scoring algorithm are freely. Statistical analysis code (SPSS syntax) is available on request.
