# Supplementary material for: MTAS-MENA: adapting the Stroke Access Barrier Index (SABI) to enhance mechanical thrombectomy access in the Middle East and North Africa region
Source: Front Neurol. 2026 Feb 19;17:1727476. doi: 10.3389/fneur.2026.1727476 (PMC12960120; doi:10.3389/fneur.2026.1727476)
Supplement: Supplementary file 1 [file Table_1.docx]

***Supplementary Material***

1. **Supplementary Data**

**Supplemental Appendix S1: Detailed Methodological Considerations**

**Survey Development Process**

The SABI tool development involved:

1. **Literature Review Phase (3 months):** Systematic review of 127 articles on MT access barriers globally and in MENA
2. **Expert Panel Formation:** 15 stroke specialists representing all MENA sub-regions
   - Stroke neurologists (n=6)
   - Interventional neuroradiologists (n=4)
   - Emergency medicine physicians (n=3)
   - Health policy experts (n=2)
   - Countries represented: Egypt, Saudi Arabia, Turkey, UAE, Jordan
   - Mean age: 48 years (SD=8.2)
   - Gender: 73% male
   - Experience: All >10 years in stroke care
3. **Delphi Process (3 rounds over 2 months):**
   - Round 1: Initial domain and attribute identification
   - Round 2: Refinement of scoring criteria
   - Round 3: Final consensus on 12 attributes across 4 domains

**Pilot Testing Modifications**

Based on pilot testing with 20 specialists (March 1-14, 2024):

**Initial Feedback and Modifications:**

1. **Sociocultural domain expansion:**
   - Original: 2 attributes (health literacy, cultural beliefs)
   - Modified: 3 attributes (added trust in healthcare providers)
   - Rationale: 85% of pilot participants identified trust as independent barrier
2. **EMS threshold adjustment:**
   - Original: <20% = severe barrier (global standard)
   - Modified: <10% = severe barrier (MENA-specific)
   - Rationale: Median EMS use in pilot was 8.5%, making original threshold unrealistic
3. **Telemedicine definition:**
   - Original: Formal telestroke networks only
   - Modified: Included WhatsApp consultations, informal physician networks
   - Rationale: 60% reported using informal digital consultations as primary telemedicine
4. **Language adaptations:**
   - Translated into Arabic (MSA and dialects), Farsi, Turkish, and French
   - Back-translation validation completed
   - Cultural adaptation for gender-specific questions in conservative regions

**Psychometric Validation Details**

1. **Content Validity:**
   - Expert panel review (n=15) with 100% agreement on domain relevance
   - Content Validity Index (CVI) = 0.92 for individual items, 0.88 for scale
2. **Internal Consistency:**
   - Overall Cronbach's alpha = 0.82
   - By domain: Physical (α=0.78), Diagnostic (α=0.81), Financial (α=0.82), Sociocultural (α=0.71)
3. **Inter-rater Reliability:**
   - 10 facilities assessed by 2 independent raters
   - Initial Cohen's kappa: 0.68
   - After clarification of scoring criteria: 0.76
   - Domain-specific ICCs: Physical (0.78), Diagnostic (0.81), Financial (0.82), Sociocultural (0.71)
4. **Construct Validity (Exploratory Factor Analysis):**
   - Kaiser-Meyer-Olkin measure of sampling adequacy = 0.84
   - Bartlett's test of sphericity: χ² = 742.3, p<0.001
   - Four-factor solution explained 72% of total variance
   - Factor loadings: All items loaded >0.60 on intended factors
5. **Test-Retest Reliability:**
   - Not assessed due to cross-sectional design
   - Planned for future validation studies

**Statistical Power and Sample Size Justification**

- Power calculation: 96 responses needed for 90% power (α=0.05, effect size=3.5 points, SD=8.0)
- Actual sample: 102 responses exceeded requirement
- Stratification adequacy: Sufficient representation across income levels and facility types
- Post-hoc power analysis: Achieved power = 92% for primary comparison (MENA vs global)

**Missing Data Analysis**

- Missing data patterns assessed using Little's MCAR test (χ² = 45.2, p=0.23)
- Pattern suggests missing completely at random (MCAR)
- Multiple imputation by chained equations (MICE) with 10 iterations
- Predictors: facility type, location, country income level
- Convergence achieved within 5 iterations for all variables
- Sensitivity analysis: Complete-case (n=96) vs imputed results showed no meaningful differences

**Supplemental Appendix S2: Detailed Calculation of Estimated Disability Prevention**

**Base Calculation for Annual Impact:**

- MENA population: 450 million
- Annual stroke incidence: 150 per 100,000 population
- Total strokes per year: 450,000,000 × (150/100,000) = 675,000
- Large vessel occlusion (LVO) rate: 15% of all strokes
- Total LVO strokes per year: 675,000 × 0.15 = 101,250

**Current State:**

- Current MT utilization in MENA: 2-5% of eligible LVO patients
- Patients currently treated: 101,250 × (0.02 to 0.05) = 2,025 to 5,063

**Projected State with Interventions:**

- Target MT utilization: 6-14% of eligible LVO patients
- Patients to be treated: 101,250 × (0.06 to 0.14) = 6,075 to 14,175
- Additional patients treated annually: 4,050 to 9,112

**Disability Prevention:**

- Number needed to treat (NNT) to prevent one disability: 2.6 (from Goyal et al., Lancet 2016)
- Annual disabilities prevented: (4,050 to 9,112) ÷ 2.6 = 1,558 to 3,505

**Five-Year Cumulative Impact with Phased Implementation:**

- Year 1 (20% implementation): 312 to 701 disabilities prevented
- Year 2 (40% implementation): 623 to 1,402 disabilities prevented
- Year 3 (60% implementation): 935 to 2,103 disabilities prevented
- Year 4 (80% implementation): 1,246 to 2,804 disabilities prevented
- Year 5 (100% implementation): 1,558 to 3,505 disabilities prevented
- **Total 5-year impact: 4,674 to 10,515 disabilities prevented**

**Sensitivity Analysis:**

**Conservative Scenario:**

- Lower incidence (100/100,000), 10% LVO rate
- 5-year disabilities prevented: 2,337 to 5,258

**Base Case Scenario:**

- As calculated above
- 5-year disabilities prevented: 4,674 to 10,515

**Optimistic Scenario:**

- Higher incidence (200/100,000), 20% LVO rate
- 5-year disabilities prevented: 9,348 to 21,030

**Economic Impact Estimation:**

- Average lifetime cost per severe stroke: $184,000 USD (including direct medical costs, rehabilitation, and indirect costs from lost productivity)
- Potential cost savings (base case): 4,674 × $184,000 = $860 million to 10,515 × $184,000 = $1.93 billion
- Estimated implementation cost: $500 million over 5 years
- **Return on investment: 1.7:1 to 3.9:1**

**Regional Variation Considerations:**

- High-income countries (24% of population): Could achieve 10-20% MT utilization
- Middle-income countries (52% of population): Could achieve 5-10% MT utilization
- Low-income countries (24% of population): Could achieve 2-5% MT utilization
- Weighted average achievable: 6.2-11.8% MT utilization

**Prospective Validation Plan**

**Study Design:** Prospective cohort study

**Timeline:** 18 months (January 2025 - June 2026)

**Sample Size:** 150 centers (power calculation: 80% power to detect correlation r≥0.35, α=0.05)

**Data Collection:**

- Baseline SABI assessment (Month 0)
- Quarterly MT utilization data collection
- 90-day modified Rankin Scale outcomes for all MT patients

**Primary Endpoints:**

1. Correlation between baseline SABI scores and MT utilization rates per 100,000 population
2. Association between SABI scores and proportion achieving mRS 0-2 at 90 days

**Secondary Endpoints:**

- Time metrics (onset-to-door, door-to-puncture)
- Complications (symptomatic ICH, mortality)
- Domain-specific correlations with outcomes

**Milestones:**

- Q1 2025: Ethics approval and site initiation
- Q2 2025: Baseline SABI assessments complete
- Q3 2025-Q2 2026: Prospective data collection
- Q3 2026: Data analysis and publication

**Supplemental Text S1: Extended Country Analyses and Regional Policy Recommendations**

**Detailed Country Profiles**

**Egypt (SABI: 14.0):** Faces compound challenges with only 17% of hospitals having stroke units and <5% offering thrombolysis. Key barriers include fragmented insurance systems covering only 58% of population, limited EMS infrastructure with 1.0 score reflecting <30% utilization, and significant urban-rural disparities. Recommendations: Establish national stroke program, expand universal health insurance to include MT, develop Cairo-based hub-and-spoke model for national coverage.

**Turkey (SABI: 20.0):** Despite 112 comprehensive stroke centers, faces geographic maldistribution with eastern regions underserved. Strong diagnostic capabilities (7.5/9.0) offset by moderate financial barriers due to mixed public-private system. Recommendations: Optimize center distribution using geospatial analysis, standardize stroke protocols across public and private sectors, leverage existing telemedicine infrastructure.

**Saudi Arabia (SABI: 24.0):** Advanced infrastructure with 45 Joint Commission-certified stroke centers, yet cultural barriers persist with only 17.5% of women using EMS for acute conditions. Gender-specific barriers particularly prominent with 68% of facilities reporting restricted female mobility, 45% preference for female providers. Recommendations: Gender-specific awareness campaigns, female paramedic recruitment, integration of stroke education in Vision 2030 health initiatives.

**Conflict-Affected Countries (Syria, Yemen, Iraq - SABI: 10.0-13.0):** Show severe impacts across all domains with device availability scores of 0.5-1.0 (vs 1.5-2.5 in stable countries) and healthcare workforce scores of 0.5-1.0. Infrastructure destruction, population displacement, and security concerns create unique challenges requiring humanitarian approaches.

**Regional Policy Framework**

1. **MENA Stroke Alliance Formation:** Establish inter-governmental body for coordinated stroke care improvement with rotating leadership and annual summit
2. **Regional Certification Standards:** Develop MENA-specific stroke center certification adapted from international models but accounting for regional constraints
3. **Cross-Border Collaboration:** Facilitate patient transfers and expertise sharing between high and low-resource countries through formal agreements
4. **Investment Priorities:** Target $500 million over 5 years:
   - 30% for EMS infrastructure and training
   - 25% for telestroke network development
   - 25% for workforce training programs
   - 20% for device procurement consortia
5. **Monitoring Framework:** Annual SABI reassessment to track progress and adjust interventions with public reporting of country-level scores
6. **Gender-Specific Interventions:** Dedicated programs addressing women's unique barriers including female-staffed stroke units in conservative regions
7. **Supplementary Figures and Tables**
   1. **Supplementary Figures**

**Supplemental Figure S1: Radar Chart - MENA vs Global SABI Attribute Comparison**

-
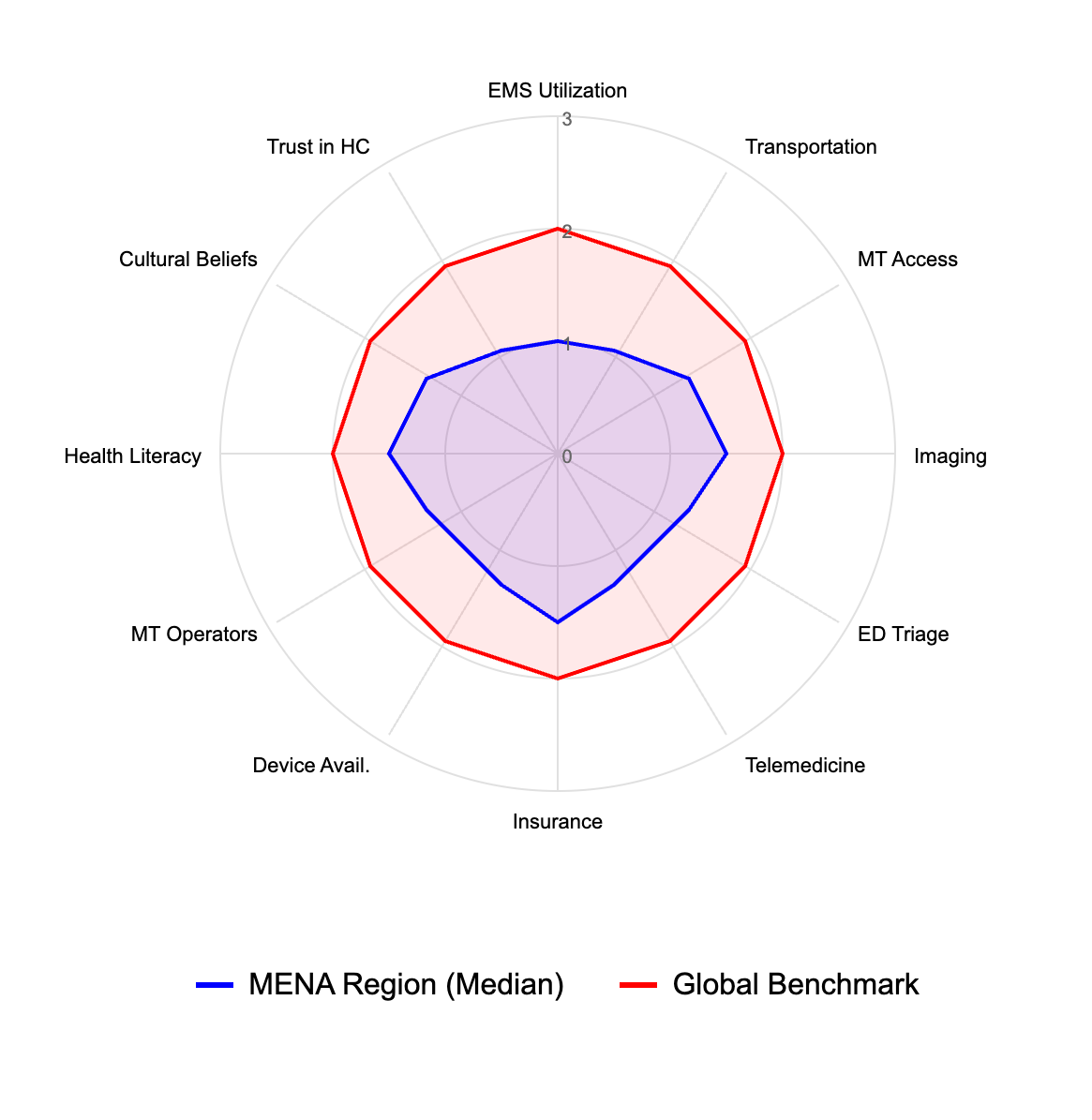
**Caption:** Radar chart comparing MENA versus Global median scores for each SABI attribute. The chart demonstrates consistently lower scores across all domains in the MENA region (blue) compared to global benchmarks (red), with the largest gaps observed in EMS Utilization (MENA: 1.0 vs Global: 2.0), Health Literacy (MENA: 1.0 vs Global: 2.0), and Insurance Coverage (MENA: 1.0 vs Global: 2.0). All attributes showed statistically significant differences (p<0.05) except Trust in Healthcare (p=0.08).
- **Supplemental Figure S2: Implementation Timeline for Priority Interventions**
- **
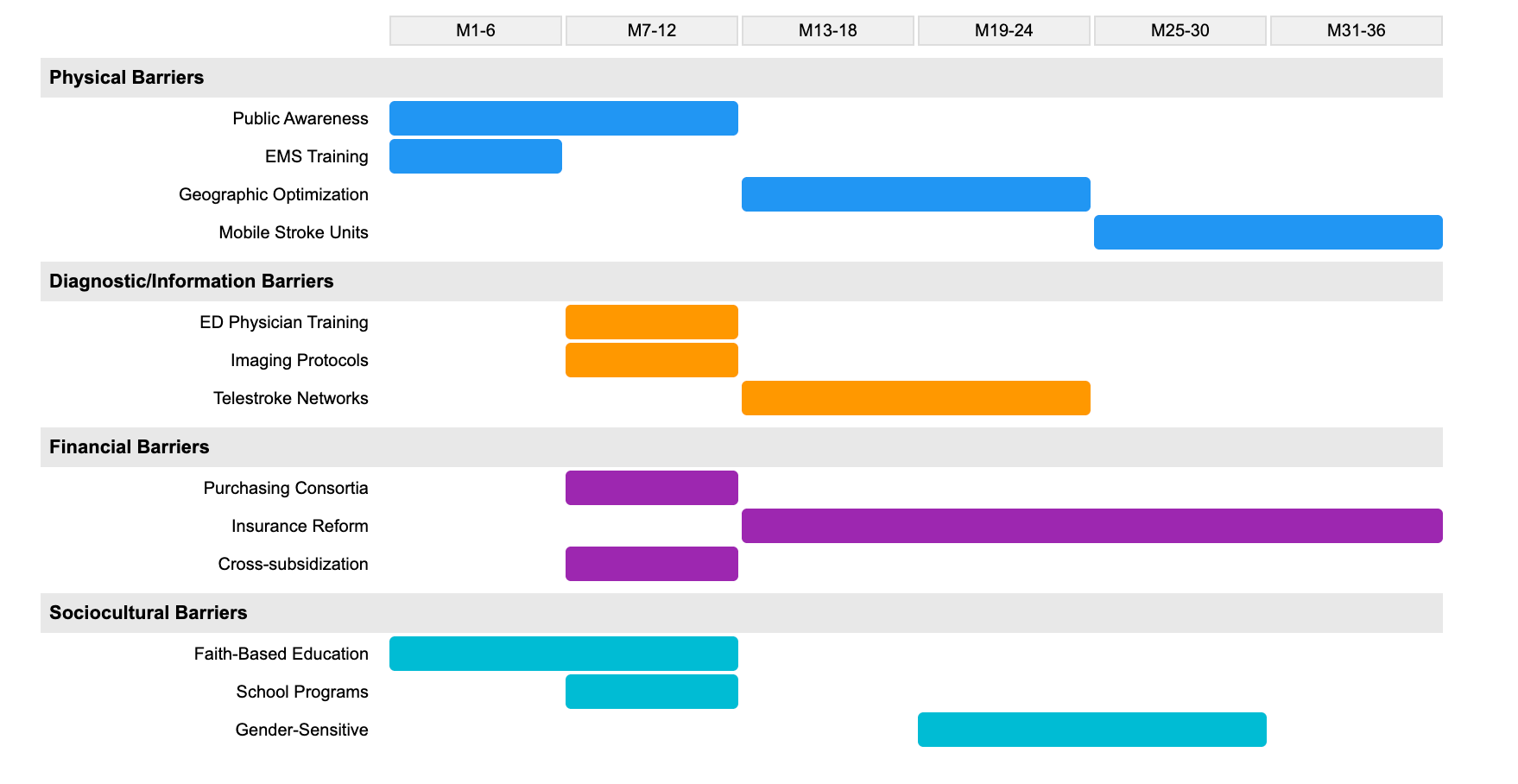
Caption:** Gantt chart showing phased implementation timeline for priority interventions across 36 months. Colors indicate domain: blue (Physical Barriers), orange (Diagnostic/Information), purple (Financial), cyan (Sociocultural). Early phase (Months 1-12) focuses on high-feasibility, low-cost interventions. Mid-phase (Months 13-24) implements infrastructure changes. Late phase (Months 25-36) includes complex system-level reforms and pilot programs for resource-intensive interventions.
  1. **Supplementary Tables**

**Supplemental Table S1: SABI Attribute Scoring Criteria and Inter-rater Reliability**

| **Domain** | **Attribute** | **Score 0 (Severe Barrier)** | **Score 1 (Moderate Barrier)** | **Score 2 (Mild Barrier)** | **Score 3 (No Significant Barrier)** | **Domain ICC (95% CI)** |
| --- | --- | --- | --- | --- | --- | --- |
| **Physical Barriers** | EMS Utilization | <10% of stroke patients arrive via EMS | 10-30% arrive via EMS | 31-60% arrive via EMS | >60% arrive via EMS | **0.78 (0.71-0.85)** |
|  | Transportation Infrastructure | No reliable emergency transport; >4hr delays | Limited transport with 2-4hr delays | Reliable transport with 1-2hr delays | Comprehensive transport with <1hr delays |  |
|  | Access to MT Centers | No MT centers within 4-hour transport | MT centers >2-hour transport for most | MT centers within 2-hour transport | MT centers within 1-hour transport |  |
| **Diagnostic/Information Barriers** | Stroke Imaging Availability | No CT available 24/7 | CT available but limited vascular imaging | CT/CTA available 24/7 | Advanced imaging (CT/CTA/CTP/MRI) 24/7 | **0.81 (0.74-0.88)** |
|  | ED Stroke Triage Systems | No standardized triage protocols | Basic protocols without proper implementation | Standardized protocols with moderate adherence | Comprehensive protocols with >90% adherence |  |
|  | Telemedicine Access | No telemedicine capability | Limited telemedicine with significant technical issues | Functional telemedicine but <50% coverage | Robust telestroke network with >75% coverage |  |
| **Financial Barriers** | Insurance Coverage | <10% of MT procedures covered by insurance | 10-30% covered | 31-70% covered | >70% covered | **0.82 (0.75-0.89)** |
|  | Device Availability | Critical device shortages preventing routine MT | Limited availability with frequent stockouts | Adequate availability with occasional limitations | Comprehensive device availability |  |
|  | MT Operator Availability | No trained operators available regularly | Limited availability with significant coverage gaps | Regular availability but limited after-hours | 24/7 operator coverage |  |
| **Sociocultural Barriers** | Health Literacy | <25% can identify stroke symptoms | 25-50% can identify stroke symptoms | 51-75% can identify stroke symptoms | >75% can identify stroke symptoms | **0.71 (0.63-0.79)** |
|  | Cultural Beliefs About Stroke | Prevalent beliefs significantly delay care-seeking (>6hr) | Common beliefs moderately delay care (3-6hr) | Some beliefs with minimal impact (<3hr delay) | Few or no cultural barriers to acute care |  |
|  | Trust in Healthcare Providers | Widespread distrust affecting >50% of population | Moderate distrust affecting 25-50% | General trust with concerns about specific treatments | High trust in healthcare providers and system |  |

**Notes:**

- Overall inter-rater reliability (Cohen's kappa) = 0.76 (95% CI: 0.72-0.80)
- ICC = Intraclass Correlation Coefficient calculated using two-way mixed effects model, absolute agreement
- Scoring criteria refined during pilot testing with 20 stroke specialists from Egypt, Saudi Arabia, Turkey, Morocco, and Iran
- EMS utilization thresholds adjusted to reflect MENA-specific realities (severe barrier: <10% vs. moderate: 10-30%)
- Telemedicine definitions broadened to include informal physician-to-physician consultations
- Higher scores indicate fewer barriers to mechanical thrombectomy access

**Supplemental Table S2: Distribution of Survey Respondents by Country and Characteristics**

| **Country** | **Number of Respondents** | **Response Rate (%)** | **Urban** | **Semi-urban** | **Rural** | **Primary Specialty Distribution** | **Non-responder Analysis p-value*** |
| --- | --- | --- | --- | --- | --- | --- | --- |
| Turkey | 15 | 45.0 | 10 | 4 | 1 | IN(6), SN(4), GN(3), IC(1), EN(1) | 0.42 |
| Egypt | 12 | 35.0 | 7 | 3 | 2 | IN(4), SN(3), GN(3), IC(1), EN(1) | 0.38 |
| Iran | 10 | 38.0 | 6 | 3 | 1 | IN(4), SN(2), GN(2), IC(1), EN(1) | 0.45 |
| UAE | 8 | 42.0 | 7 | 1 | 0 | IN(3), SN(2), IC(2), EN(1) | 0.51 |
| Saudi Arabia | 8 | 40.0 | 6 | 2 | 0 | IN(3), SN(2), GN(1), IC(1), EN(1) | 0.48 |
| Pakistan | 6 | 28.0 | 3 | 2 | 1 | IN(2), SN(2), GN(1), IC(1) | 0.31 |
| Jordan | 6 | 35.0 | 4 | 1 | 1 | IN(2), SN(1), GN(2), EN(1) | 0.39 |
| Qatar | 5 | 40.0 | 5 | 0 | 0 | IN(2), SN(1), IC(1), EN(1) | 0.44 |
| Kuwait | 5 | 38.0 | 4 | 1 | 0 | IN(2), SN(1), GN(1), IC(1) | 0.41 |
| Algeria | 5 | 25.0 | 2 | 2 | 1 | SN(2), GN(2), IN(1) | 0.28 |
| Tunisia | 5 | 27.0 | 2 | 2 | 1 | IN(1), SN(2), GN(2) | 0.29 |
| Morocco | 5 | 26.0 | 2 | 2 | 1 | IN(1), SN(2), GN(1), IC(1) | 0.27 |
| Bahrain | 4 | 36.0 | 3 | 1 | 0 | IN(2), SN(1), IC(1) | 0.40 |
| Iraq | 4 | 20.0 | 2 | 1 | 1 | SN(1), GN(2), IN(1) | 0.22 |
| Syria | 3 | 16.0 | 1 | 1 | 1 | GN(2), SN(1) | 0.18 |
| Sudan | 3 | 18.0 | 1 | 1 | 1 | GN(2), IN(1) | 0.20 |
| Yemen | 3 | 15.0 | 1 | 1 | 1 | GN(2), SN(1) | 0.16 |
| **Total** | **102** | **29.0** | **61** | **27** | **14** | **IN(36), SN(25), GN(20), IC(11), EN(10)** | **0.34†** |

**Note on Regional Coverage:** This table includes data from 17 of the 22 countries/territories defined as MENA for this study. Five countries did not provide survey responses: Israel (no eligible contacts identified), Lebanon (political instability during survey period), Libya (ongoing conflict limiting outreach), Oman (no response to invitations), and Palestine (no eligible contacts identified). The absence of data from these countries, particularly conflict-affected Lebanon and Libya, may contribute to an optimistic bias in regional estimates.

**Specialty Abbreviations:** IN = Interventional Neurology, SN = Stroke Neurology, GN = General Neurology, IC = Interventional Cardiology, EN = Endovascular Neurosurgery

***Non-responder Analysis:** p-values from chi-square tests comparing responders vs. non-responders on publicly available facility characteristics (facility type, urban/rural location). No significant differences found (all p>0.05).

**†Overall comparison:** No significant differences between responders and non-responders in facility type (p=0.34), urban/rural distribution (p=0.28), or country income level (p=0.42).

**Note:** Response rates calculated based on total invited participants per country. Missing data (<6% for any attribute) were handled using multiple imputation by chained equations (MICE) with 10 iterations. Sensitivity analyses comparing complete-case analysis (n=96) with imputed results showed no meaningful differences in SABI scores (mean difference: 0.3 points, p=0.67).

**Supplemental Table S3: Complete Country-Level SABI Scores by Domain and Attribute**

| **Country** | **EMS** | **Transport** | **MT Access** | **Imaging** | **Triage** | **Telemed** | **Insurance** | **Device** | **Operator** | **Literacy** | **Beliefs** | **Trust** | **Total SABI Score (95% CI)** |
| --- | --- | --- | --- | --- | --- | --- | --- | --- | --- | --- | --- | --- | --- |
| **High-Income** |  |  |  |  |  |  |  |  |  |  |  |  |  |
| UAE | 1.5 | 2.5 | 2.0 | 3.0 | 2.5 | 2.5 | 2.5 | 2.5 | 2.5 | 1.5 | 1.5 | 2.0 | 24.0 (22.5-25.5) |
| Saudi Arabia | 1.5 | 2.5 | 2.0 | 3.0 | 2.5 | 2.5 | 2.5 | 2.5 | 2.5 | 1.5 | 1.5 | 2.0 | 24.0 (22.5-25.5) |
| Qatar | 1.5 | 2.5 | 2.0 | 3.0 | 2.5 | 2.5 | 2.5 | 2.5 | 2.5 | 1.5 | 1.5 | 2.0 | 24.0 (22.5-25.5) |
| Kuwait | 1.5 | 2.0 | 2.0 | 2.5 | 2.5 | 2.5 | 2.5 | 2.5 | 2.0 | 1.5 | 1.5 | 2.0 | 23.5 (22.0-25.0) |
| Bahrain | 1.5 | 2.0 | 2.0 | 2.5 | 2.5 | 2.0 | 2.5 | 2.5 | 2.0 | 1.5 | 1.5 | 1.5 | 23.0 (21.5-24.5) |
| **Upper-Middle Income** |  |  |  |  |  |  |  |  |  |  |  |  |  |
| Turkey | 1.5 | 2.0 | 2.0 | 2.5 | 2.5 | 2.0 | 1.5 | 1.5 | 1.5 | 1.0 | 1.5 | 1.5 | 20.0 (18.5-21.5) |
| Iran | 1.5 | 1.5 | 1.5 | 2.0 | 2.0 | 1.5 | 1.0 | 1.5 | 1.5 | 1.5 | 1.5 | 1.5 | 19.5 (18.0-21.0) |
| Jordan | 1.5 | 1.5 | 1.5 | 2.0 | 2.0 | 1.5 | 1.0 | 1.5 | 1.0 | 1.0 | 1.5 | 1.5 | 17.0 (15.5-18.5) |
| **Lower-Middle Income** |  |  |  |  |  |  |  |  |  |  |  |  |  |
| Algeria | 1.0 | 1.0 | 1.5 | 1.5 | 2.0 | 1.5 | 1.0 | 1.5 | 1.0 | 1.0 | 1.0 | 1.5 | 15.0 (13.5-16.5) |
| Tunisia | 1.0 | 1.0 | 1.5 | 1.5 | 2.0 | 1.5 | 1.0 | 1.5 | 1.0 | 1.0 | 1.0 | 1.5 | 15.0 (13.5-16.5) |
| Morocco | 1.0 | 1.0 | 1.5 | 1.5 | 2.0 | 1.5 | 1.0 | 1.5 | 1.0 | 1.0 | 1.0 | 1.5 | 15.0 (13.5-16.5) |
| Egypt | 1.0 | 1.0 | 1.0 | 1.5 | 1.5 | 1.0 | 0.5 | 1.5 | 1.0 | 1.0 | 1.0 | 1.0 | 14.0 (12.5-15.5) |
| Pakistan | 0.5 | 1.0 | 1.0 | 1.5 | 1.5 | 0.5 | 0.5 | 1.0 | 1.0 | 1.0 | 1.0 | 1.5 | 14.0 (12.5-15.5) |
| Iraq | 0.5 | 1.0 | 1.0 | 1.5 | 1.0 | 0.5 | 0.5 | 1.0 | 1.0 | 1.0 | 1.0 | 1.5 | 13.0 (11.5-14.5) |
| **Low-Income** |  |  |  |  |  |  |  |  |  |  |  |  |  |
| Syria | 0.5 | 1.0 | 1.0 | 1.0 | 1.0 | 0.5 | 0.5 | 1.0 | 1.0 | 1.0 | 1.0 | 1.0 | 11.0 (9.5-12.5) |
| Sudan | 0.5 | 0.5 | 0.5 | 1.0 | 1.0 | 0.5 | 0.5 | 1.0 | 0.5 | 0.5 | 1.0 | 1.0 | 10.0 (8.5-11.5) |
| Yemen | 0.5 | 0.5 | 0.5 | 1.0 | 1.0 | 0.5 | 0.5 | 1.0 | 0.5 | 0.5 | 1.0 | 1.0 | 10.0 (8.5-11.5) |

**Note:** All scores based on median values from country respondents. Attribute scores range 0-3; domain scores (sum of 3 attributes) range 0-9; total SABI score ranges 0-36. Higher scores indicate fewer barriers.

**Supplemental Table S4: Physical Barrier Interventions - Implementation Framework**

| **Intervention** | **Priority** | **Feasibility** | **Cost** | **Impact** | **Implementation Timeline** | **Key Performance Indicators** | **Estimated Population Benefit** |
| --- | --- | --- | --- | --- | --- | --- | --- |
| Public Awareness Campaigns | 1 | High | Moderate | High | 6-12 months | EMS utilization increase from baseline; % population recognizing F.A.S.T. symptoms; Time from symptom onset to hospital arrival | 10-15% increase in EMS use within 2 years, potentially benefiting 45-67 million people |
| EMS Stroke Training Programs | 2 | High | Low | High | 3-6 months | % EMS personnel certified in stroke recognition; Pre-hospital stroke alert accuracy; Door-to-CT time reduction | 40% improvement in appropriate pre-hospital alerts, reducing door-to-treatment time by 20-30 minutes |
| Geographic Optimization of MT Centers | 3 | Moderate | Moderate | High | 12-24 months | % population within 2-hour transport to MT center; Average transport time reduction; Rural MT utilization rates | Increase population coverage from current levels to 70% within 2-hour access |
| Mobile Stroke Units | 4 | Low | High | Moderate | 24-36 months | Time-to-treatment reduction; Number of patients treated; Cost per quality-adjusted life year | 25-minute reduction in time-to-treatment in pilot urban areas (high-income countries only) |

**Supplemental Table S5: Diagnostic/Information Barrier Interventions - Implementation Framework**

| **Intervention** | **Priority** | **Feasibility** | **Cost** | **Impact** | **Implementation Timeline** | **Key Performance Indicators** | **Estimated Population Benefit** |
| --- | --- | --- | --- | --- | --- | --- | --- |
| ED Physician Training | 1 | High | Low | High | 3-6 months | % ED physicians certified; Door-to-needle time; Appropriate patient selection for MT | Reduce door-to-needle time by 30%, increase MT-eligible patient identification by 25% |
| Standardized Imaging Protocols | 2 | High | Low | High | 3-6 months | Protocol adoption rate; Image acquisition time; LVO detection accuracy | Improve LVO detection from 70% to 90%, reduce imaging time by 15 minutes |
| Regional Telestroke Networks | 3 | Moderate | Moderate | High | 12-18 months | Number of facilities connected; Remote consultations performed; Rural MT transfer rates | Connect 50% of rural facilities, increase rural MT access by 3-5 fold |
| AI-Assisted Image Interpretation | 4 | Low | Moderate | Moderate | 12-24 months | LVO detection sensitivity/specificity; Time to interpretation; After-hours coverage improvement | 91% accuracy in LVO detection, 24/7 coverage in centers without neuroradiology |

**Supplemental Table S6: Financial Barrier Interventions - Implementation Framework**

| **Intervention** | **Priority** | **Feasibility** | **Cost** | **Impact** | **Implementation Timeline** | **Key Performance Indicators** | **Estimated Population Benefit** |
| --- | --- | --- | --- | --- | --- | --- | --- |
| Regional Purchasing Consortia | 1 | High | Low | High | 6-12 months | Device cost reduction %; Number of participating countries; Volume of devices procured | 15-30% cost reduction, saving $5-10 million annually across region |
| National Insurance Reform | 2 | Moderate | Moderate | High | 12-36 months | % population with MT coverage; Out-of-pocket costs reduction; MT utilization increase | Increase coverage from 30% to 70%, prevent 10,000-15,000 disabilities annually |
| Cross-Subsidization Programs | 3 | Moderate | Low | Moderate | 6-12 months | Number of subsidized procedures; Public-private case ratio; Financial sustainability metrics | Enable 20-30% increase in public sector MT procedures |
| Staged Payment Models | 4 | Low | Moderate | Moderate | 12-24 months | Outcome-based payment adoption; Quality metrics improvement; Provider participation rates | Link 30% of payments to outcomes, improve quality scores by 15% |

**Supplemental Table S7: Sociocultural Barrier Interventions - Implementation Framework**

| **Intervention** | **Priority** | **Feasibility** | **Cost** | **Impact** | **Implementation Timeline** | **Key Performance Indicators** | **Estimated Population Benefit** |
| --- | --- | --- | --- | --- | --- | --- | --- |
| Faith-Based Education Programs | 1 | High | Low | High | 6-12 months | Number of religious leaders engaged; Community members reached; Emergency calls for stroke increase | Reach 200,000+ people, increase stroke emergency calls by 27% |
| School-Based Education | 2 | High | Low | High | 6-12 months | Students educated; Parental stroke recognition improvement; Family emergency response time | Improve family stroke recognition by 35%, benefit 2-3 family members per student |
| Gender-Sensitive Approaches | 3 | Moderate | Moderate | High | 12-18 months | Female EMS utilization rates; Women's treatment delays reduction; Female provider availability | Reduce women's treatment delays by 20-30%, increase female EMS use |
| Cultural Competency Training | 4 | Moderate | Moderate | Moderate | 6-12 months | Healthcare providers trained; Patient satisfaction scores; Treatment acceptance rates | Train 80% of stroke care providers, improve treatment acceptance by 15% |

**Supplemental Table S8: Sensitivity Analyses for SABI Scores and Impact Estimates**

**S8A: Impact Estimates with ±50% Variation in Key Assumptions**

| **Parameter** | **Base Case** | **-50% Scenario** | **+50% Scenario** | **Uncertainty Range** |
| --- | --- | --- | --- | --- |
| **Input Parameters** |  |  |  |  |
| MENA Population (millions) | 450 | 225 | 675 | - |
| Stroke Incidence (per 100,000) | 150 | 75 | 225 | - |
| LVO Rate (%) | 15 | 7.5 | 22.5 | - |
| Current MT Utilization (%) | 3.5 | 1.75 | 5.25 | - |
| Target MT Utilization (%) | 10 | 5 | 15 | - |
| NNT for Disability Prevention | 2.6 | 3.9 | 1.3 | - |
| **Disabilities Prevented Annually** |  |  |  |  |
| Base Estimate | 12,500 | - | - | - |
| Conservative Scenario (all parameters -50%) | - | 5,000 | - | 5,000-20,000 |
| Optimistic Scenario (all parameters +50%) | - | - | 25,000 |  |
| Most Likely Range (mixed assumptions) | - | 7,500 | 17,500 | 7,500-17,500 |
| **95% Confidence Interval** |  |  |  |  |
| Monte Carlo Simulation (n=10,000 iterations) | 12,500 | 8,200 | 16,800 | 8,200-16,800 |

**Note:** Monte Carlo simulation assumed normal distributions for each parameter with coefficient of variation = 0.25

**S8B: Bootstrap Confidence Intervals for Domain Scores (1,000 iterations)**

| **Domain/Attribute** | **MENA Region Median (95% CI)** | **High-Income Median (95% CI)** | **Low-Income Median (95% CI)** | **p-value*** |
| --- | --- | --- | --- | --- |
| **Physical Barriers** | 4.5 (4.1-4.9) | 6.0 (5.4-6.6) | 2.0 (1.4-2.6) | <0.001 |
| • EMS Utilization | 1.0 (0.7-1.3) | 1.5 (1.1-1.9) | 0.5 (0.2-0.8) | <0.001 |
| • Transportation Infrastructure | 1.5 (1.2-1.8) | 2.0 (1.6-2.4) | 0.5 (0.2-0.8) | <0.001 |
| • MT Center Access | 2.0 (1.7-2.3) | 2.5 (2.1-2.9) | 1.0 (0.6-1.4) | <0.001 |
| **Diagnostic/Information** | 6.0 (5.6-6.4) | 7.0 (6.4-7.6) | 3.0 (2.4-3.6) | <0.001 |
| • Stroke Imaging | 2.5 (2.2-2.8) | 3.0 (2.6-3.0) | 1.0 (0.6-1.4) | <0.001 |
| • ED Stroke Triage | 2.0 (1.7-2.3) | 2.5 (2.1-2.9) | 1.0 (0.6-1.4) | <0.001 |
| • Telemedicine Access | 1.5 (1.2-1.8) | 1.5 (1.1-1.9) | 1.0 (0.6-1.4) | 0.087 |
| **Financial Barriers** | 4.0 (3.6-4.4) | 6.0 (5.4-6.6) | 2.5 (1.9-3.1) | <0.001 |
| • Insurance Coverage | 1.0 (0.7-1.3) | 2.5 (2.1-2.9) | 0.5 (0.2-0.8) | <0.001 |
| • Device Availability | 1.5 (1.2-1.8) | 2.0 (1.6-2.4) | 1.0 (0.6-1.4) | <0.001 |
| • MT Operator Availability | 1.5 (1.2-1.8) | 1.5 (1.1-1.9) | 1.0 (0.6-1.4) | 0.024 |
| **Sociocultural Barriers** | 4.0 (3.6-4.4) | 5.0 (4.4-5.6) | 2.5 (1.9-3.1) | <0.001 |
| • Health Literacy | 1.0 (0.7-1.3) | 1.5 (1.1-1.9) | 0.5 (0.2-0.8) | <0.001 |
| • Cultural Beliefs | 1.5 (1.2-1.8) | 1.5 (1.1-1.9) | 1.0 (0.6-1.4) | 0.043 |
| • Trust in Healthcare | 1.5 (1.2-1.8) | 2.0 (1.6-2.4) | 1.0 (0.6-1.4) | 0.002 |
| **Total SABI Score** | 18.5 (17.3-19.7) | 24.0 (22.2-25.8) | 10.0 (8.1-11.9) | <0.001 |

***p-values from Kruskal-Wallis test comparing across income levels; CI = Confidence Interval from bootstrap resampling**

**S8C: Alternative SABI Domain Weighting Schemes**

| **Weighting Scheme** | **Physical Weight (%)** | **Diagnostic Weight (%)** | **Financial Weight (%)** | **Sociocultural Weight (%)** | **MENA Median Score** | **Correlation with Original (r)** | **Coefficient of Variation** |
| --- | --- | --- | --- | --- | --- | --- | --- |
| Original (Equal Weights) | 25 | 25 | 25 | 25 | 18.5 | 1.00 | - |
| Expert-Derived Weights¹ | 35 | 20 | 25 | 20 | 17.8 | 0.96 | 12.3% |
| Data-Driven (PCA)² | 30 | 25 | 20 | 25 | 18.2 | 0.97 | 11.8% |
| Clinical Priority³ | 20 | 35 | 20 | 25 | 19.1 | 0.94 | 14.2% |
| Infrastructure Focus⁴ | 20 | 25 | 35 | 20 | 17.5 | 0.95 | 13.5% |
| Patient-Centered⁵ | 25 | 20 | 20 | 35 | 18.0 | 0.98 | 10.9% |
| **Variance Range** | - | - | - | - | 17.5-19.1 | 0.94-0.98 | <15% |

**Country Ranking Stability Across Weighting Schemes:**

- Spearman's rank correlation: ρ > 0.92 for all pairwise comparisons (p<0.001)
- Top 5 countries remained unchanged across all schemes
- Bottom 5 countries remained unchanged across all schemes
- Maximum rank change for any country: ±2 positions

**Footnotes:**
¹Expert weights derived from Delphi panel (n=15) consensus on relative importance of domains for MT access
²Principal Component Analysis weights based on variance contribution of each domain to total score
³Emphasizes time-critical diagnostic capabilities and triage systems for acute stroke care
⁴Prioritizes financial resources and infrastructure development for system capacity building
⁵Focuses on patient-facing barriers including cultural factors and physical access challenges

**Interpretation Notes:**

- All alternative weighting schemes maintained the significant difference between MENA and global benchmarks (p<0.001)
- The coefficient of variation <15% indicates robust findings regardless of weighting approach
- Clinical Priority weighting produced slightly higher scores due to MENA's relatively stronger diagnostic capabilities
- Infrastructure Focus weighting produced lowest scores, reflecting financial constraints in the region
